# Developing the architecture and characteristics for a green hydrogen ecosystem to effectively accelerate the energy transition: a contribution to innovation leadership

**DOI:** 10.1007/s11356-025-36092-1

**Published:** 2025-03-20

**Authors:** Daniel Schwappach, Werner G. Faix, Jens Mergenthaler, Claus-Christian Carbon

**Affiliations:** 1 Department of General Psychology and Methodology, University of Bamberg, Otto-Friedrich-Universitat Bamberg, Markusplatz 3, 96047 Bamberg, Bavaria Germany; 2 School of International Business and Entrepreneurship (SIBE), Steinbeis University, Kalkofenstr. 53, 71083 Herrenberg, Baden-Wuerttemberg, Germany

**Keywords:** Ecosystem, Hydrogen, Innovation, Leadership, Management, Climate change, **Code**: O13, O3, P48, Q21, Q28, Q4

## Abstract

Green hydrogen from renewable resources is one of the most critical levers for counteracting global warming caused by anthropogenic greenhouse gas emissions and, at the same time, increasing energy security. Green hydrogen is about to move from an early innovation stage to an industrial scale. Leaders can shape this transition using ecosystem theory. We used an exploratory mixed-methods study design to investigate the architecture of such an ecosystem with actors and the characteristics with objectives, roles, and key activities. We interviewed in the first step 22 experts using a semi-structured interview guide and facilitated in the second step a focus group discussion with 24 participants to test the insights gained from the expert interviews for their practicality. The data analyzed by qualitative content analysis revealed four main actor segments sufficient to describe participation in the green hydrogen ecosystem (GHE). The focus group discussion adds a fifth group, which could be described as the central expert council actor segment, which optimizes the processes between the actors, emphasizing that all actor segments are pursuing a common objective, the decarbonization under the Paris Agreement from 2015. Three actor segments in the ecosystem are identified as leaders to realize the common objective: equipment and service providers, governments and authorities, and the hydrogen market. The subjective perception of a low return on investment, considering the efforts an actor needs to contribute to the joint value creation and the achievement of the actor’s individual objectives, is with the actor segments with the leadership responsibility. In the medium to long term, this could lead to tensions and an imbalance in the ecosystem, which could be mitigated by a more transparent distribution and allocation of key activities in proportion to the achievement of objectives.

## Introduction

Based on the 2015 Paris Agreement, adopted by 195 countries, more than 90 countries have set a goal to decarbonize their country completely to net zero (Schumer et al. 2023; European Commission 4/22/2022). The European Union (EU) has set a goal of achieving net-zero decarbonization by 2050 (European Commission 2019). Low-carbon or carbon-free hydrogen is one of the main pillars of decarbonization to net-zero (Kouchaki-Penchah et al. 2023). In addition to the net-zero targets for carbon exhaust, the ongoing conflict in Ukraine since 2022 has accelerated demand for natural gas alternatives—especially in Germany (bdew 2022; European Commission 4/22/2022).

By September 2022, more than 40 countries have published hydrogen roadmaps and strategies (Hydrogen Council and McKinsey & Company 2022b). It is expected that between 175 and 420 GW of capacity to produce hydrogen by electrolysis using electricity with low to no greenhouse gas emissions as the energy source will be realized and commissioned by the end of the decade (International Energy Agency 2023). Of these, nearly 700 MW (0.3%) is installed and in operation by 2022 and more than 2 GW by 2023. Fifty-eight percent of projects are in the early feasibility phase, and 38% are in the feasibility phase. A financial investment decision has been made for 3% of the projects. The average size of projects in operation is 12 MW, which can be considered as initial pilot projects. By 2025, the average size will increase to 100–500 MW and up to 1 + GW by 2030 (Aurora Energy Research 2021). The cost of hydrogen based on renewable energy is relatively high at US$5.4–4/kg compared to US$1–1.8/kg for hydrogen with a carbon footprint in 2020 (Burgess 2022; Heid et al. 2022). The consumption of low-carbon hydrogen represents less than 1% of global consumption (International Energy Agency 2023). Certain technologies along the value chain for decarbonized hydrogen are more at an innovation stage and require further development. The figures indicate that the step from governmental targets to concrete measures has been carried out. Most of the production of decarbonized hydrogen is currently foreseen to be realized with technologies such as water electrolysis. Those technologies have limited projects in operation, with rather limited size and volume. The production costs and technology developments have challenges to deal with, and the entire ecosystem must make the step from pilot innovation to the commercial business phase (Schwappach et al. 2024).

Leaders must lead human communities into an innovative and creative future under framework conditions (Faix et al. 2020). Human communities mean, in other words, ecosystems and framework conditions (*Umwelt*^1^ (environment)). For bringing green hydrogen and its derivates to large-scale application and realization, the actors within the ecosystem (human communities) and the *Umwelt* with its trends (framework conditions) must be guided into an innovative and creative future. Hydrogen innovations that are driven forward in a socio-technical system context require clarity on the part of the social subsystem with its actors and structures (Griffiths et al. 2021; Hester 2014). The holistic view of the green hydrogen ecosystem (GHE) and its environment seems to be underrepresented in scientific research (Schwappach et al. 2024; Gordon et al. 2023). The definition of actors and the associated characteristics of the GHE are not well understood. These aspects are important to guide the leaders of such a GHE to make decisive progress in developing a green hydrogen economy at scale. It will enable the transition from the innovation phase to the business phase and ultimately meet society’s demand for decarbonization. The present paper aims to answer the research question of what the definitions of the architecture with actors and actor segments and the characteristics with objectives, roles, and key activities of the GHE are. The paper of Dedehayir et al. (2018) provides the theoretical foundation for role and key activity definitions. Our paper adds a case study about the transition of ecosystems from innovation to business phase (Dedehayir et al. 2018). It will provide the basis for the explanations on the behavioral process of the GHE.

## Background of hydrogen ecosystems

This section presents background information on hydrogen ecosystems by starting with general information about hydrogen and then taking a deeper look into the ecosystem literature.

### Hydrogen

Three predominant colors are used to define hydrogen carbon footprint (Gribova and Giese 2021). The focus of this study is on green hydrogen produced by water electrolysis.

- Gray hydrogen is from natural gas through steam reforming with greenhouse gas emissions.
- Blue hydrogen is from natural gas through steam reforming with reduced greenhouse gas emissions through carbon capture, utilization, and storage. It is often considered an enabler of the energy transition (Cavalcante et al. 2024).
- Green hydrogen can be produced by water electrolysis using electricity with little or no greenhouse gas emissions. Other alternatives for production are thermal energy, biochemical energy, or photonic energy (Dincer 2012; Borges et al. 2024).

Two main factors are driving the development of hydrogen (Gielen et al. 2019). First, an increasing number of countries have taken action to meet their decarbonization targets based on the 2015 Paris Agreement (Hydrogen Council and McKinsey & Company 2022b). Paradoxically, these targets could initially lead to an increase in greenhouse gas emissions, as the majority of today’s hydrogen is gray, as a study in Australia shows (Qadeer et al. 2023). Second, the production cost for green hydrogen is expected to decrease from US$5.4–4.0/kg in 2020 to US$2.0/kg in 2030 (Burgess 2022; Heid et al. 2022). Green hydrogen is used to convert electricity from electrons into molecules that can be stored for months and, in some cases, transported around the world using existing infrastructure (Alsaba et al. 2023). This makes green hydrogen an attractive and affordable energy carrier, regardless of the location in which it is used.

Growth rates for hydrogen produced by electrolysis vary between the institutions that provided the figures and the year in which the statistics were published. This uncertainty is, for instance, reflected in the wide range of 175 to 420 GW given by the International Energy Agency (2023). The Hydrogen Council (2023) noticed that up to 230 GW capacity will be operational in 2030. However, in 2022, 700 MW of electrolysis was installed and in operation (International Energy Agency 2023). The deviation of the potential-installed hydrogen capacity from electrolysis shows that it is still at an early stage with uncertainties about the actual capabilities of implementation. The obstacles to achieving a predictable growth rate are the uncertainty about the cost of large-scale hydrogen production, infrastructure investment, mass storage, transportation, safety issues, uncertainties in supply and offtake agreements, and the limited capacity of renewable electricity installed (Clerici and Furfari 2021; Eljack and Kazi 2021).

Europe appears to be the frontrunner with 35% or USD 117 billion of the world’s announced projects (Hydrogen Council and McKinsey & Company 2023). In the initial phase, the hydrogen economy is dependent on government funding unless the monetary benefits are considerable enough for the private sector (Kar et al. 2022). At EUR 10.6 billion, Europe provided the most funding for projects under its Important Projects of Common European Interest funding program, followed by the US Reduction Inflation Act. Nevertheless, an investment gap along the hydrogen value chain of USD 380 billion by 2030 exists. By 2030, the highest hydrogen consumption is expected in China (29%), Europe (18%), and North America (18%) (Hydrogen Council and McKinsey & Company 2022a). These countries will be the leading producers, although imports will be necessary. Imports to the main consumer markets will come from derivatives such as ammonia, steel, and methanol from the Middle East, Australia, and South America (Hydrogen Council and McKinsey & Company 2022a). Hydrogen will be used for various industrial applications such as oil refining, ammonia, fertilizer, and steel, mobility applications with methanol for shipping and e-fuels for aviation and for power generation applications for flexible power generation (Kearney Energy Transition Institute 2020).

### Ecosystems

**Fig. 1:**
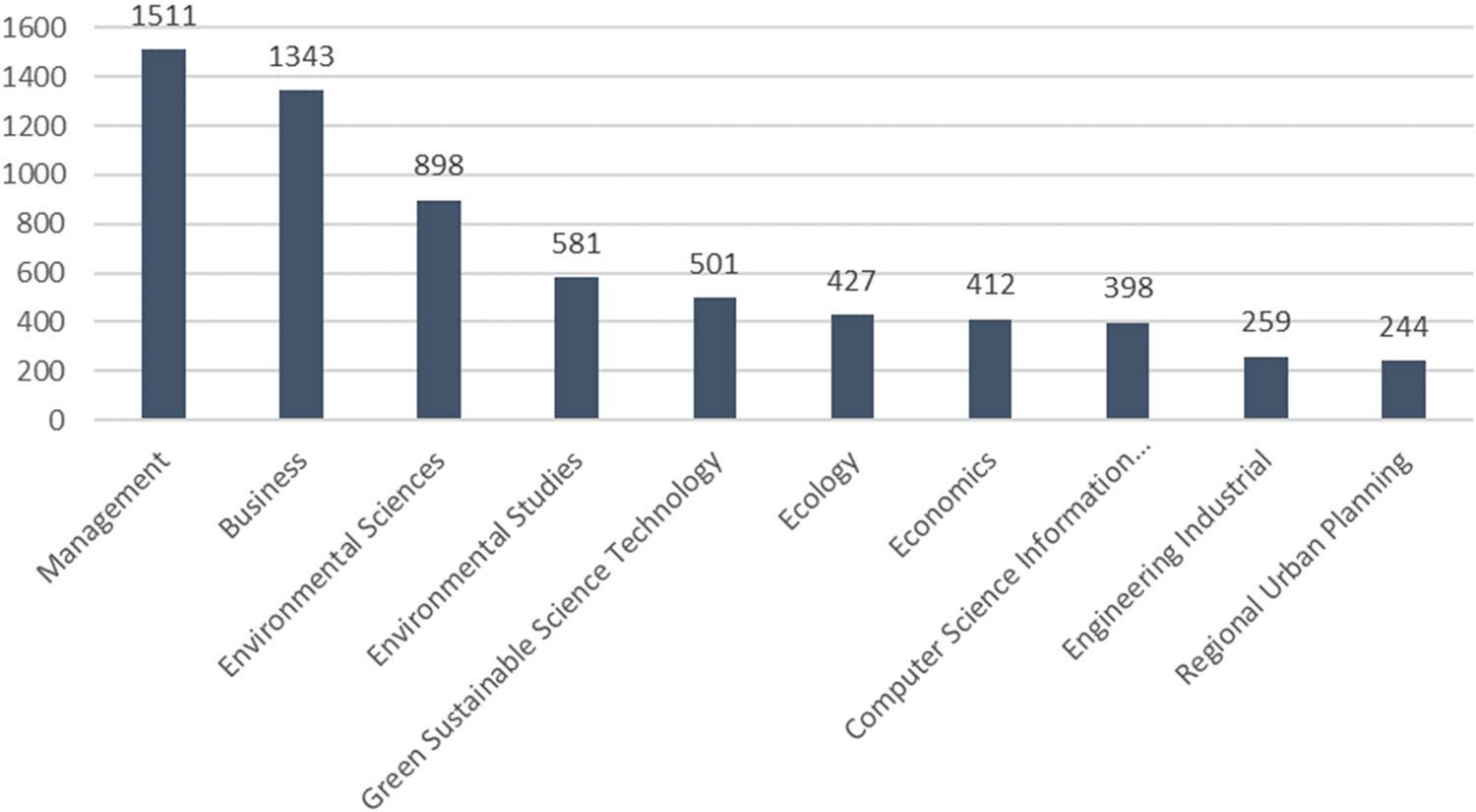
Publication of ecosystems research. Citation data are derived from Clarivate Web of Science, copyright Clarivate 2024. All rights reserved

In the article “Predators and Prey,” Moore (1993) marked the beginning of ecosystem theory in a business context. The subject has grown more popular. In 2015, already 39 articles for the combination of the terms on ecosystems, business, and innovation were published on the Web of Science abstract platform (search term: ‘ecosystem*’ AND ‘business*’ AND ‘innovation*’) (Scaringella and Radziwon 2018). Forty-seven new articles were added in 2015 and 2016. Scaringella and Radziwon (2018) included 393 references to ecosystems in their theoretical systematic literature review. The search for ((ALL = (ecosystem)) AND ALL = (business)) AND ALL = (innovation) with Web of Science on October 7, 2023, shows 5490 results. Overall, it can be assumed that there is a clearly observable increase in interest on ecosystem theories regarding business and innovation (Fig. 1).

Moore (1993) enumerated four evolutionary stages of corporate success: birth, expansion, leadership, and self-renewal. Examples such as IBM reveal that the ecosystem is critical to both the progress and decline of a company in a sector. Through co-evolution, ecosystem actors can thrive together. In some cases, a place in the ecosystem can be secured through patents. Continuous innovation is an important factor in being an active actor within an ecosystem. The leadership role is unique because it is “…the Fight for Control in an Ecosystem” (Moore 1993, p. 80). The leadership role provides the opportunity to shape the ecosystem with its needs to best fit the leader’s business/product by continuously initiating rapid innovation for improvement.

Russell and Smorodinskaya (2018) discussed the similarities and differences between innovation ecosystems and systems. Based on current economic patterns, the triple helix should be implemented as ecosystems. “Institutionally different sectors, representing the business sector, the knowledge-generating sector (universities, research institutes, other R&D centers), and the public sector (government agencies or authorities)” are the identified actor segments (Russell and Smorodinskaya 2018, p. 117). The paper contains considerations for governance of an innovation ecosystem.

The characteristics of business innovation ecosystems can be described using ecological examples (Shaw and Allen 2018). That helps to underscore the importance of pathways of interconnected business models. Along such paths, matter, information, and resources are transformed into value. This recycles scarce resources such as customer attention and customer-generated information. The *Umwelt* as a self-world controls the transformation process by influencing the business model, as it has an individual influence on each participant, but it also limits the transformation. The business model of the ecosystem participants reflects the influence of the *Umwelt* and indicates membership in the ecosystem.

Dedehayir et al. (2018) described the roles within an innovation ecosystem focusing on the birth. Four main segments are defined: “…leadership roles (‘ecosystem leader’ and ‘dominator’), direct value creation roles (‘supplier’, ‘assembler’, ‘complementor’, and ‘user’), value creation support roles (‘expert’ and ‘champion’), and entrepreneurial ecosystem roles (‘entrepreneur’, ‘sponsor’, and ‘regulator’)…” (Dedehayir et al. 2018, p. 18). Additionally, they defined specific activities carried out by the individual group members. Furthermore, the paper indicates the multiple roles of each ecosystem participant that can change over the progress of the innovation.

Tsujimoto et al. (2018) systematically reviewed available scientific ecosystem research. They proposed an ecosystem multi-actor network with a central focus on business ecosystems. It has been mentioned that there is an involvement of the innovation that leads to changing roles of the participants and the participations itself. Understanding the patterns behind the rationality and decision principles of each participants helps outline the reasons for a topic’s growth or decline and ecosystems. It is outlined that there could be patterns in the behavioral chain which are not recognized by the actors. Such patterns could affect the growth or the decline of ecosystem.

In a literature review on ecosystem boundaries, Cobben et al. (2022) provided an overview of which ecosystems enable the implementation of ecosystem archetype–specific goals. The four ecosystem archetypes, knowledge, innovation, entrepreneurship, and business, were investigated. The boundaries they identified “…are in seven dimensions of the ecosystem types: (1) competitive advantage, (2) geographical scope, (3) ecosystem development, (4) orchestration, (5) actor types and roles, (6) structure and (7) value creation and capture” (Cobben et al. 2022, p. 149).

## Methodology

### Research design

**Fig. 2:**
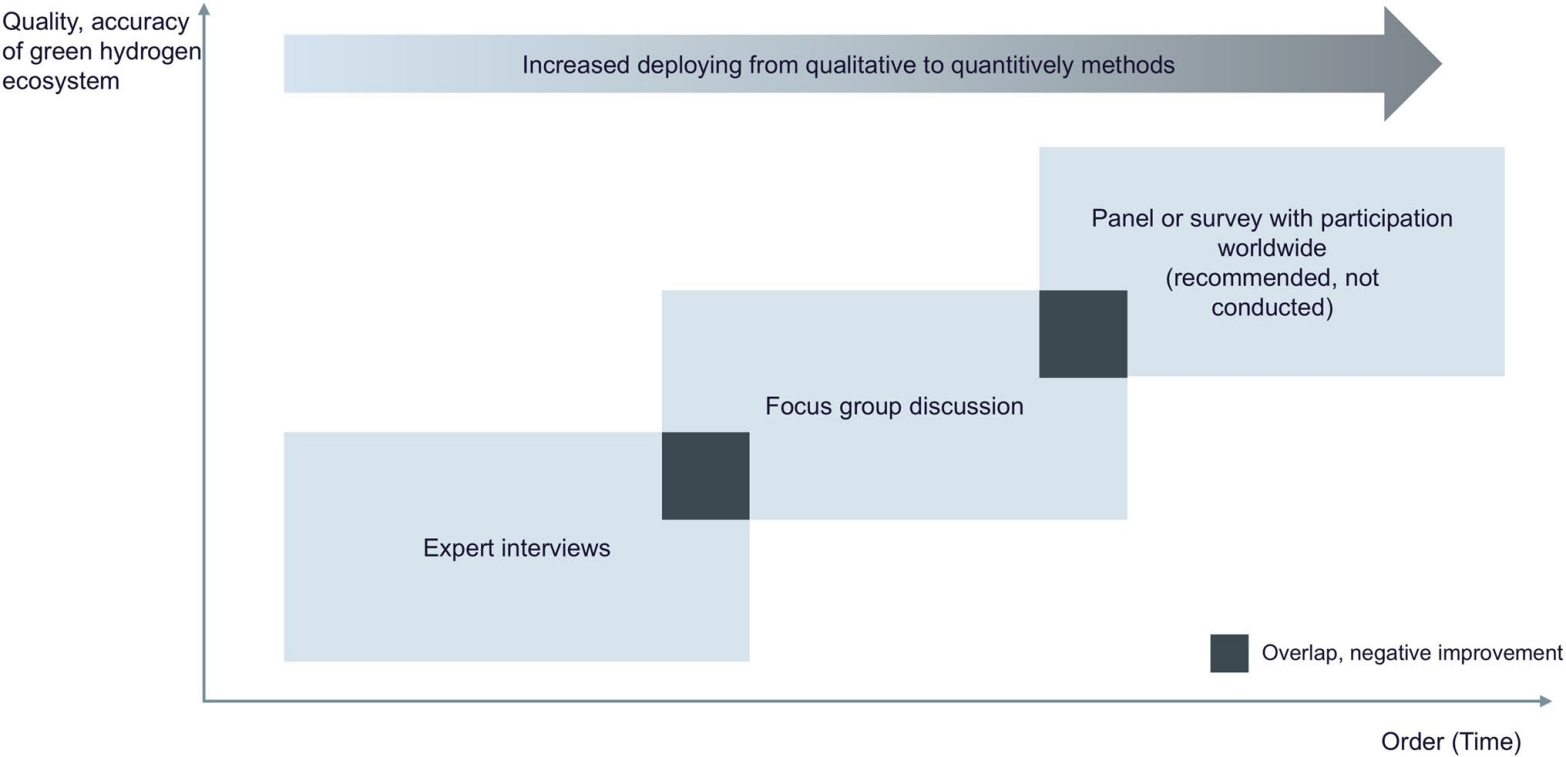
Research plan for exploring the green hydrogen ecosystem (GHE)

We found only a limited number of applications of ecosystem theories specifically to the hydrogen sector in the scientific literature. We used an exploratory mixed-methods study design for this paper to capture the human experience holistically (Mey and Mruck 2010). We focus on architecture and characteristics as these are required to define the behavior of the GHE. The method triangulation of qualitative and quantitative analyses aims to combine the strengths and mitigate weaknesses of both methods (Denzin 1970). The combination of methods is used for validation or validation criticism and leads to complementary, congruent, or divergent research results (Baur and Blasius 2014). It is required to analyze the results for convergence, divergence, and complementary (Mey and Mruck 2010). In the subsequential order of the research collection, it should improve the quality and accuracy of the GHE, as visualized in Fig. 2. For an explorative research approach, qualitative research methods could be deployed and provide the base for quantitative research with a more extensive coverage. The mixed-methods approach leaks a commonly agreed-upon systematic taxonomy for typical method problems and validation risks. The expert interview and the focus group discussion were selected as research methods. For both methods, a mixed-methods approach was selected to capture qualitative patterns and validate pre-prepared assumptions by the frequency of mentionings. The focus group discussion as the second applied method is used to test insights from the expert interviews (Nyumba et al. 2018). It can be considered a kind of reality and practical proof of scientifically determined patterns.

### Expressions

An exploratory discussion can benefit from a theory outline in exemplifying and visualization of an illustration (Gracht and Kisgen 2022). Creating an illustration is an intermediate step and the basis for expressions and vice versa (Glaser et al. 2008). In this study, two expressions are examined.

#### Expression 1: four main segments of actor groups with science and research, hydrogen market, equipment and service providers, and governments and authorities are engaged in the green hydrogen ecosystem 

This paper further explores the views on the actor segments of the GHE. The triple helix ecosystem consideres three segments of actors (2018), the triple helix ecosystem includes at least three segments of actors. The business group appears to be many actors along the hydrogen value chain with a wide range of objectives (Schwappach et al. 2024). The GHE joint value creation focuses on the optimal price for green hydrogen, which returns to capital expenditures and operating costs. The equipment and service providers target the investment cost, while the operating costs are the focus of the plant owner and operator (Hydrogen Council and McKinsey & Company 2021). In addition, it can be assumed that there will be local and global markets for the supply and offtake of green hydrogen (Griffiths et al. 2021). The hydrogen and derivatives market has its own objectives to achieve and key activities to contribute to the joint value creation. It was decided to split the business group into the two segments of actors: equipment and service providers as well hydrogen market (incl. derivatives). This study outlines the knowledge creation segment as science and research. Government and authorities stand for the public sector. The research for the actor segments is independent of organizational sizes such as small- and medium-sized enterprises.

#### Expression 2: each actor segment is characterized by individual objectives, roles, and key activities, and the leader(s) have the governance visible through their objectives, roles and key activities

The different actor segments will be described by certain characteristics: objectives, roles, and key activities. The objectives of the actor segment should be in line with the joint value creation (Dedehayir et al. 2018). The objectives at an ecosystem level are for innovation and business ecosystems, among others, mainly derived from the leader objectives (Cobben et al. 2022). That means if the objective of the ecosystem is known, it can be linked to the objectives of the leading actor segments. Since the major objective of the GHE is decarbonization, it needs to be the objective of the leader actors, too. Objectives combined with the ecosystem partners’ competencies and ideas result in a joint value proposition (Cobben et al. 2022). In an ecosystem, there are different roles to be fulfilled to shape a stable system. Having the objectives and roles identified, key activities will be the resulting consequences. For instance, Dedehayir et al. (2018) worked out a comprehensive overview of key activities linked to the roles. These terms for roles and key activities form the basis of the illustration and the related question for the expert interviews. Managing uncertainties is one of the critical activities of all actors within an ecosystem (Vasconcelos Gomes et al. 2018).

### Illustration

**Fig. 3:**
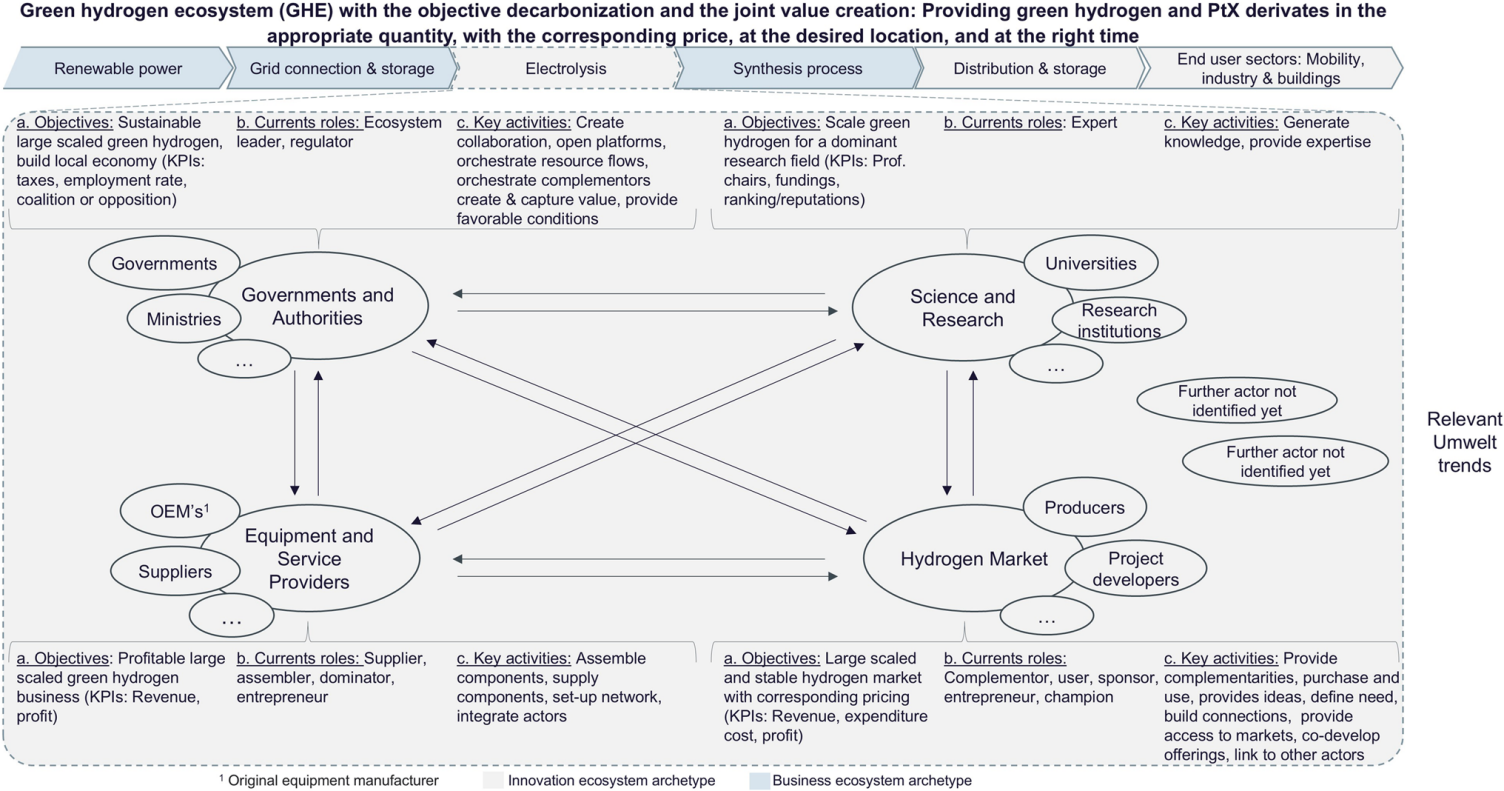
Green hydrogen ecosystem (GHE) illustration based on previous publication of the authors and pre-selected objectives, current roles, and key activities

GHE illustration (Fig. 3) summarizes the status and serves as a foundation for further analysis in this paper. We derived it from our previous work on GHE (Schwappach et al. 2024). The GHE illustration with the objective of decarbonization has the predefined joint value creation “providing green hydrogen and…” and PtX^2^ derivatives “…in the appropriate quantity, with the corresponding price, at the desired location, and at the right time” (Schwappach et al. 2024, p. 30). The segmentation is considered along the value chain, introduced after the interviews and the focus group discussion, and the transition from innovation to the business ecosystem status. Renewable power, grid connection, and storage and synthesis process can be considered in the business ecosystem archetype because they are state of the art (Alhanaee et al. 2023; Jones et al. 2021; Griffiths et al. 2021). Even if the technologies involved are state of the art, the use of renewable energies such as wind and solar brings several challenges that need to be overcome, for example, direct use of renewable electricity vs. use for hydrogen production, what is the best mix of wind and solar power, and how much excess capacity is required to ensure some continuous operation over a year, especially in the case of derivative production of ammonia where reactor start-up and shutdown increases the degradation, and the issue of grid stabilization, especially in remote areas in island operation, requires additional investments such as synchronized condensers and battery storage (Gielen et al. 2019; L’Huby et al. 2020; Bora et al. 2024; Pereira et al. 2017). The availability of electricity and its volatility influences the selection of the electrolyzer, as technologies such as proton-exchange membrane (PEM) have faster ramp and start-up rates than alkaline (Nasser et al. 2022; Shiva Kumar and Himabindu 2019). As the technologies are available, the focus is more on the techno-economic optimization of the overall system (Nasser et al. 2022; Zhou et al. 2024; Zhang and Zhou 2024). For the synthesis process, e.g., Haber–Bosch or Fischer–Tropsch, it does not matter whether the hydrogen is green or gray. The electrolysis, distribution and storage, and end-use sectors are more dependent on technology in an innovation ecosystem. Some midstream and downstream technologies such as electrolyzers (e.g., CAPEX decrease), distribution and storage (liquid organic hydrogen carriers, ammonia crackers, effective storage, ports, pipelines), and end-user sectors with mobility, industry, and buildings need to be newly developed or reviewed and modified (Harichandan and Kar 2023; Chen et al. 2023; Liu et al. 2020). For instance, the catalytic performance of the metal alloys used for water electrolysis is still being researched in order to improve the efficiency of these technologies (Xu et al. 2023). The direct storage and transportation of hydrogen is still cost-intensive, and some materials still do not meet the requirements of security for transportation (Liu et al. 2020). Those value chain technologies are considered in the innovation archetype stage. The GHE shows four actor segments, which include diverse actors. For each of the segments of actors, the objectives, current roles, and key activities were pre-selected by the authors. The current role and key activities were taken from the framework of Dedehayir et al. (2018) and assigned by the authors. In the adopted framework, four main categories are defined. Leadership is the first with ecosystem leader and dominator as roles. Direct value creation considers four roles: supplier, assembler, complementor, and expert and champion, followed by the category value support with expert and champions. Finally, there is an entrepreneurial ecosystem with the roles of entrepreneur, sponsor, and regulator.

### Expert interviews

The qualitative content analysis methodology proposed by Mayring (2015) was chosen to analyze the expert interviews. Ten direct and four guiding questions were generated by the expressions and the illustration (Table 7 in the Appendix). Depending on the data available upfront, the guiding questions can be either inductive or deductive in nature. The interviews were facilitated by using the pre-established GHE illustration to provide a shared understanding. The information was divided into several slides. The coding rules were defined by descriptions of Mayring (2015), which included expressions, questions, a description of the rule, instances for the codes, analysis results, aims, the observations of both assessors, and testing for quality metrics.

The sample size for expert interviews is widely discussed. The expected amount of data points ranges between 15 and 100 (Clayton 1997; Warth et al. 2013). The final number for a study depends on the feasibility. The limited number of resources, such as time, meets an infinite number of potential interviewees. For the four actor segments, it seems reasonable to interview five persons for each group. Besides belonging to one of the actor segments, the interviewees must be involved in the hydrogen sector. The expert group was part of the first author’s network. The selected interviewees were approached via LinkedIn or email. Due to delayed commitments, 22 persons were interviewed instead of 20 (Table 8 in the Appendix). Governments and authorities are overrepresented by seven instead of five. We considered all 22 persons interviewed to capture subjective opinions as much as possible. The authors highlight any relevant bias effect caused by this partial imbalance. Amberscript (Amberscript Global B.V.) was used to assist in transcribing the recordings. MAXQDA Plus software (by VERBI) was used to categorize and analyze the qualitative data. The categories were pre-inserted, and the appropriate text passages were marked for guiding questions using a deductive approach. For the inductive guiding questions, the text passages were marked first, and the categories were created following the conclusion of the 22 interviews. Two individuals independently coded the transcripts using the coding guidelines. Both sets of findings were recorded in the coding rules and compared in various meetings. Differences between the two evaluators, such as missing statements or misinterpretations, were noted and discussed, and in every instance, a value was agreed upon. The number of mentionings for the expert interview is shown in the “Results” section.

We are unable to objectify the analysis completely for the reasons listed.

- The respondents have unique perspectives. With one exception, all respondents are employed in Germany.
- The evaluators have an impact on both the survey and the evaluation.

The first point belongs to the term sampling. As earlier described, we aimed to reach a balance for the amount of participants from each actor segment. As there are two additional participants in the governments and authorities actor segment, we have highlighted shifts in the “Results” section. The last mentioned point belongs to the interviewer effect (Baur and Blasius 2014). To mitigate the impact of the interviewer, a semi-structured interview guide was used to ensure a consistent sequence of questions. In addition, the pretest was conducted to ensure the correct handling of the technical equipment, establishing contact with the interviewee, addressing objections, and ensuring the basics of the standardized survey. To increase the quality of the evaluation, the evaluation was carried out independently by two persons and subsequently compared. The standard deviation is visualized in the graphics by error bars indicating ± 1 standard deviation (SD). We are confident that the study can draw a comprehensive picture of the GHE, even after accounting for these potential biases.

### Focus group discussion

Focus group discussion methodology is, in general, counted to the qualitative methods according to Odag and Schreier in Mey and Mruck (2010). They are used to determine attitudes and thoughts through the interaction between the participants in a friendly and open environment, according to Vogl in Baur and Blasius (2014). The advantage of focus group discussion is that the participants need to formulate and argue their opinions that lead to a collective narrative instead of personal (and, thus, subjective) opinions. The disadvantage is that the group dynamic could lead to the adaption mechanism of personal opinions to group or leader opinions. For the evaluation qualitative analysis, procedures are used, especially qualitative content analysis, according to Mayring (2015). The focus group discussion was held on the premises of Leibniz Universität Hannover as part of the hydrogen expert and leader training of the three intuitions Carl von Ossietzky University Oldenburg, in cooperation with Leibniz Universität Hannover and the Fraunhofer Institute for Wind Energy Systems IWES on September 17, 2022 (link to training: https://uol.de/weiterbildung-wasserstoff). According to Eifler in Baur and Blasius (2014), the sample group can be considered a real group. There were 24 participants. All participants completed a study and have multiple years of professional experience in branches like energy economy, chemical industry, process plant construction, banking, consulting, and public authorities. To a certain extent, the participants are actors within the GHE, as shown in Fig. 8.

To introduce the topics to the participants, a presentation based on the insights gained from the expert interviews was prepared. The GHE was split into three topics: actors and objectives, roles and leadership, as well as activities and operations. Leadership and operations are not further considered in this paper.

1. Group of actors and objectives
  - How much do you see yourself with one of the actors or actor segments? (Scale 0 low, 7 very strong)
  - Are there any objectives to be added/changed?
2. Group of roles and leadership
  - How would you rank the actors for the leadership role? (Ranking 1 highest, 11 lowest)
  - Are there any actor roles to be added/changed?
3. Group of activities and operations
  - What could be done differently?
  - Are there key activities and operations to be added/changed?

As a basis, the results of the expert interviews were considered for the illustration and shared with the focus group participants. The 24 participants were divided into six teams, two teams for one topic. The participants worked on the results in the teams for about 30 min. The author and the trainer of the training visited the teams to clarify questions of understanding and moderate the discussion. Afterward, the results for each topic were presented and discussed by the teams in plenary within 20 min. Due to time constraints, not all teams could show their entire results. The presentations were recorded, and the results were documented with pictures. The authors evaluated the material.

In terms of the quality of the results, the focus group discussion challenges and adds to the insights gained by the expert interviews. The sample group can be considered as too large. Baur and Blasius (2014) suggest group sizes of six to ten (max. 12). The time for proper moderation and discussion within the teams was limited, and not all teams were able to present their results. The frequency of mentionings could not be determined due to the dynamics of the discussion, and the results of one group are not representative of the opinion of all participants. Even some participants have a GHE actor background, not everyone was able to fully follow the discussion. However, the additional findings contribute to the GHE picture and are therefore considered in the following section.

## Results

We interviewed 22 experts with a semi-structured interview guide. We worked with 24 participants of a focus group discussion to gain further insights adding to the description of the architecture and characteristics of the GHE. The compiled data are provided in this section of the paper. The authors sorted the comments into the most relevant categories for the “Objectives,” “Roles,” and “Key activities” sections. This ensures that all outcomes are included in the study. The key activities match the roles mentioned. The authors have ensured that in cases where a role was mentioned but not associated with key activity, the role was subsequently listed and vice versa. This provides consistency between the roles and the key activities for expert interviews and focus group discussions. In the case of content overlaps between the proposed objectives, roles, and key activities and the mentionings made by the participants in the expert interviews and the focus group discussion, the responses were categorized as congruent. The mentionings were categorized into additional remarks in the case of supplementary responses by the expert interview participants compared to those proposed. Mentionings from the focus group discussion that are not part of the proposed objectives, roles, or key activities and were not additionally mentioned by the experts in the interview were categorized as additional. The additional comments have been retained to give more background to the original mentioning. The wording was adjusted where necessary for better understanding, and the roles and key activities were transferred to the predefined vocabulary, according to Dedehayir et al. (2018). A defined terminology is crucial for shaping constructivist perceptions of reality. This ensures comparability and enables the discussion.

### Expert interviews

#### Architecture

The objective of the section “Architecture” is to identify actors and approve the predefined actor segments. The first question is inductive to identify the potential actors. The second question is deductive to assign the mentioned actors to the predefined actor segments if possible. Multiple answers were possible. Table 1 shows the key terms mentioned, the frequency of mentionings, the framed categories, and the assigned predefined actor segment. Actors belonging to the hydrogen market are most mentioned. The mentioned actors draw up the entire value chain. That is in line with the gained insight for the segmentation.

**Table 1:** Actors and assignment to predefined actor segments based on expert interviews

Actors mentioned by expert interviews	Mentionings	Actor categories	Assigned actor segments
Original equipment manufacturer	23	Equipment manufacturer	Equipment and service providers
Solution provider, e.g., engineering, procurement, and construction (EPC)			
Life cycle service provider			
Suppliers			
Bus manufacturer			
Society (all)	19	Offtake	Hydrogen market
Households			
Mobility (trains, garbage trucks, buses)			
Industry as consumers			
Government	17	Government and authorities	Governments and authorities
Authorities (ministries)			
Gas network operator	16	Transport	Hydrogen market
Storage			
Transporter			
Technical gas companies (processors)			
Producers (with own land)	14	H_2_ producers	Hydrogen market
Energy company as producer			
Oil and gas companies as producers			
Research institutions	12	Science and research institutions	Science and research
Universities			
Electricity supplier	12	Power and municipal utilities	Hydrogen market
Grids			
Electricity producer			
Water supplier			
Project developers	7	Project developer	Hydrogen market
Engineering offices			
Banks	6	Investors	Hydrogen market
Investors			
Platforms	2	Certifiers	Hydrogen market
Certifiers			
Traders	2	Traders	Hydrogen market
Marketers on the exchange			

#### Objectives

**Fig. 4:**
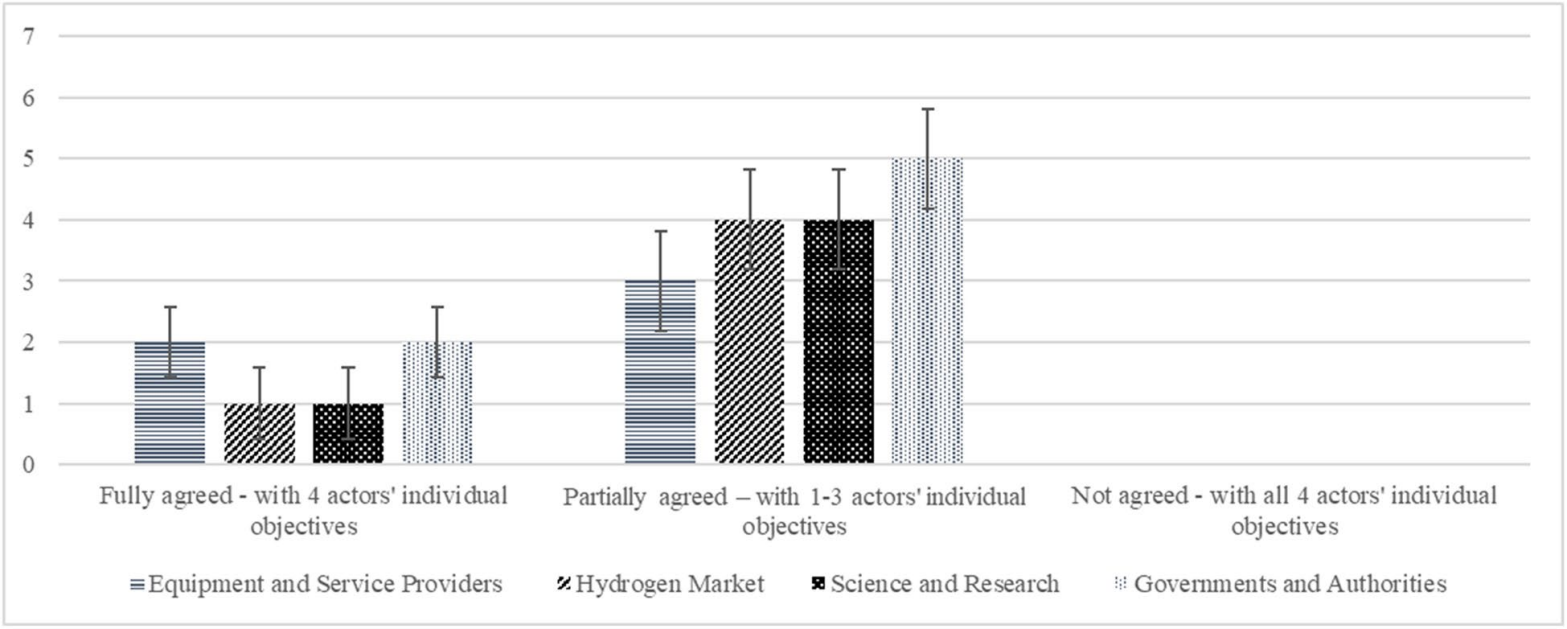
Agreement with predefined objectives. Error bars indicate ± 1 SD

The experts were asked about the application of the predefined objectives for the segments of actors. The illustration with predefined objectives was shared during the interview. The deductive approach was converted from an optional to an inductive one in case of disagreement to identify additional objectives to be added. Figure 4 shows that with 16 mentionings, there is mainly partial agreement. There is complete agreement with six mentionings. The distribution between the actors is not decisive.

The answers to the inductive optional question about the objectives to be added or changed can be found in Table 2. The remarks are divided into congruent and additional remarks, as explained in the introduction to the “Results” section. Decarbonization is mentioned for all actor segments. It can be seen as an overarching objective. There were additional remarks for all four actor segments.

**Table 2:** Actor objectives and remarks by experts and focus group discussion participants

Assigned actor segment	Proposed objectives	Remarks on objectives by expert interviews (number of mentionings)	Remarks on objectives mentioned by focus group discussion
Equipment and service providers	Profitable large-scale green hydrogen business (KPIs: revenue, profit)	Additional: - Decarbonization (1) - Market strength, high market shares (1)	Congruent: - Decarbonization Additional: - Technological leadership
Science and research	Scale green hydrogen for a dominant research field (KPIs: Prof. chairs, fundings, ranking/reputations)	Additional: - Decarbonization (1) - Make the world a better place (2)	Congruent: - Decarbonization Additional: - Development of hydrogen and PtX workforce
Governments and authorities	Sustainable large-scale green hydrogen, build local economy (KPIs: taxes, employment rate, coalition, or opposition)	Additional: - Decarbonization (1) - Number of projects realized (1)	Congruent: - Decarbonization Additional: - Social acceptance - Regional value creation
Hydrogen market	Large-scale and stable hydrogen market with corresponding pricing (KPIs: revenue, expenditure cost, profit)	Congruent: - Making money (2) Additional: - Decarbonization (9)	Congruent: - Decarbonization
*KPIs *key performance indicators			

**Fig. 5:**
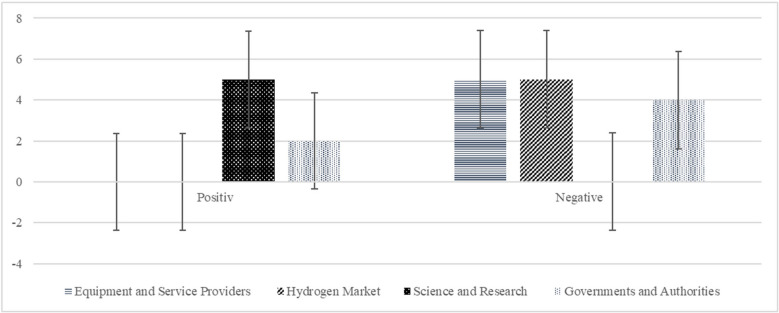
Return of investment estimation for objective achievement. Error bars indicate ± 1 SD. One abstention

To evaluate the degree of objective achievement compared to the contribution to joint value creation, the experts were asked about the return on investment. Investments are seen as contributions in the form of the key activities to the joint value creation. This is not necessarily about a financial investment but rather about the formulated objectives. The deductive question thus has no scale since the purposes are different. Rather, it captures the subjective impression through a positive or negative evaluation. As shown in Fig. 5, science and research see its investment return as positive. Governments and authorities are partially favorable, with two mentionings, four negative and one abstention. The hydrogen market and equipment and service providers assess their return of investment as negative.

#### Roles

**Fig. 6:**
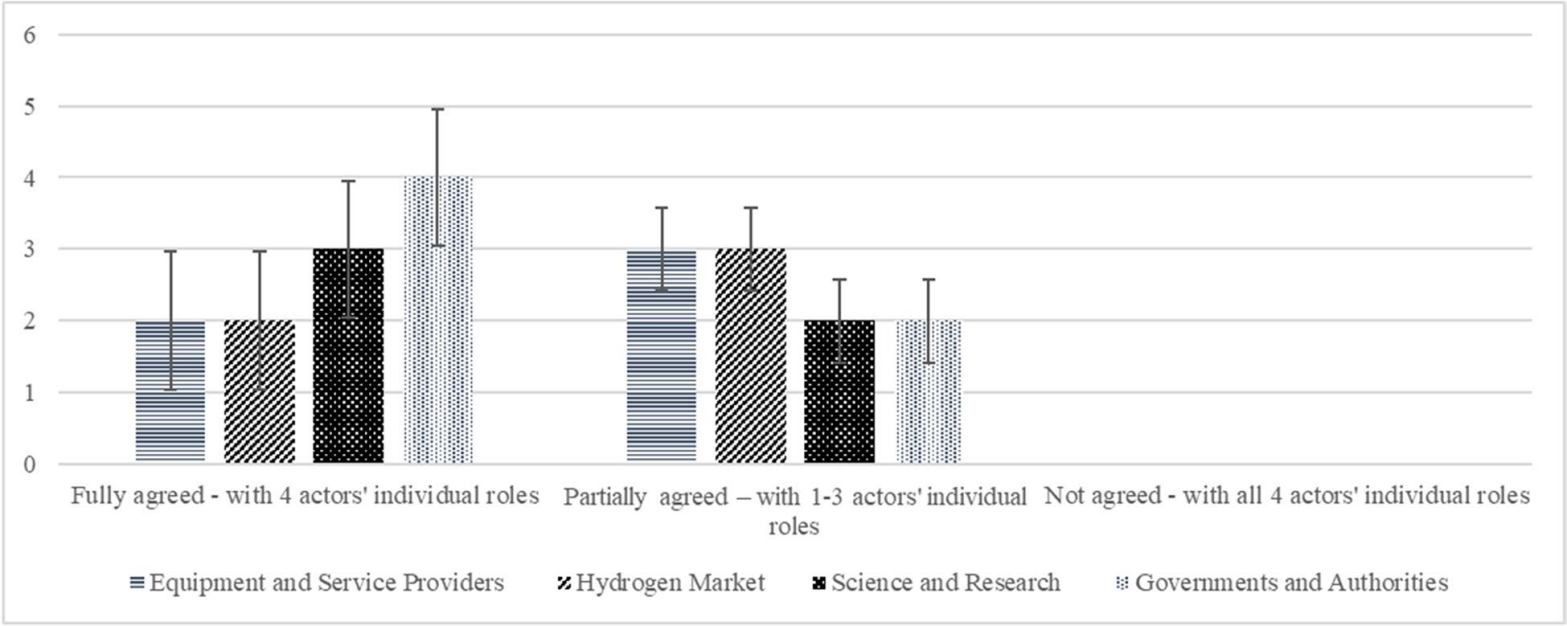
Agreement with predefined roles. Error bars indicate ± 1 SD. One abstention

To identify the roles of the actor segments, the experts were asked along the predefined roles whether they fully agree, partially agree, or disagree. The deductive approach is followed by an inductive, optional question to identify further roles. Figure 6 shows that 11 experts agreed with the predefined roles and 10 agreed in part. No decisive distribution between the different segments of actors is discernible. There is one abstention.

The congruent and additional proposed roles resulting from the second optional question are listed in Table 3. The actor segment equipment and service providers and hydrogen market are also seen as leaders. Governments and authorities are pre-classified as leaders. Science and research are not seen in a leading role. For equipment and services providers, there are additional remarks, experts, and complementors. Sponsor, dominator, and user are added to government and authorities. In addition to the ecosystem leader, the dominator for the hydrogen market was mentioned.

**Table 3:** Actor roles remark by expert interview and focus group discussion participants

Assigned actor segment	Proposed roles	Remarks on roles by expert interviews (number of mentionings)	Remarks on roles by focus group discussion
Equipment and service providers	- Supplier - Assembler - Dominator - Entrepreneur	Congruent: - Dominator (1) Additional: - Expert (1) - Complementor (1) - Ecosystem leader (2)	Congruent: - Complementor: energy provider, certifier and norming, transporter, and processing of H_2_ and PtX end product - Assembler: manufacturer of equipment - Ecosystem leader: orchestrate resource flows in the actor segment - Entrepreneur: international network activities Additional: - Sponsor: banking (financing of projects) - Champion: workflow moderation between the actor segments and central coordination
Science and research	- Expert	Congruent: - Expert	Congruent: - Expert
Governments and authorities	- Ecosystem leader - Regulator	Congruent: - Ecosystem leader (1) Additional: - Sponsor (2) - Dominator (1) - User (1)	Congruent: - Ecosystem leader: market-based incentives (e.g., grants, contract of differences, ensuring green energy on low-cost base, regional and global development of joint value creation, communication to society) show advantages (e.g., qualification and future jobs) and disadvantages - Regulator: attraction of investments, implement faster approval procedure
Hydrogen market	- Complementor - User - Sponsor - Entrepreneur - Champion	Congruent: - Champion (1) - Complementor (1) - Champion: integrator with project developer (1) - User: leverage large-scale application for cheap mass production (1) - Sponsor: leveraging small-scale solutions for initiation and learning (1) Additional: - Dominator (1) - Ecosystem leader (4)	Congruent: - User: producer, consumer - Champion: traders - Sponsor: investor, project developer
Additional: central expert council (mentioned by focus group discussion)			Additional: Champion: intermediary role

#### Key activities

**Fig. 7:**
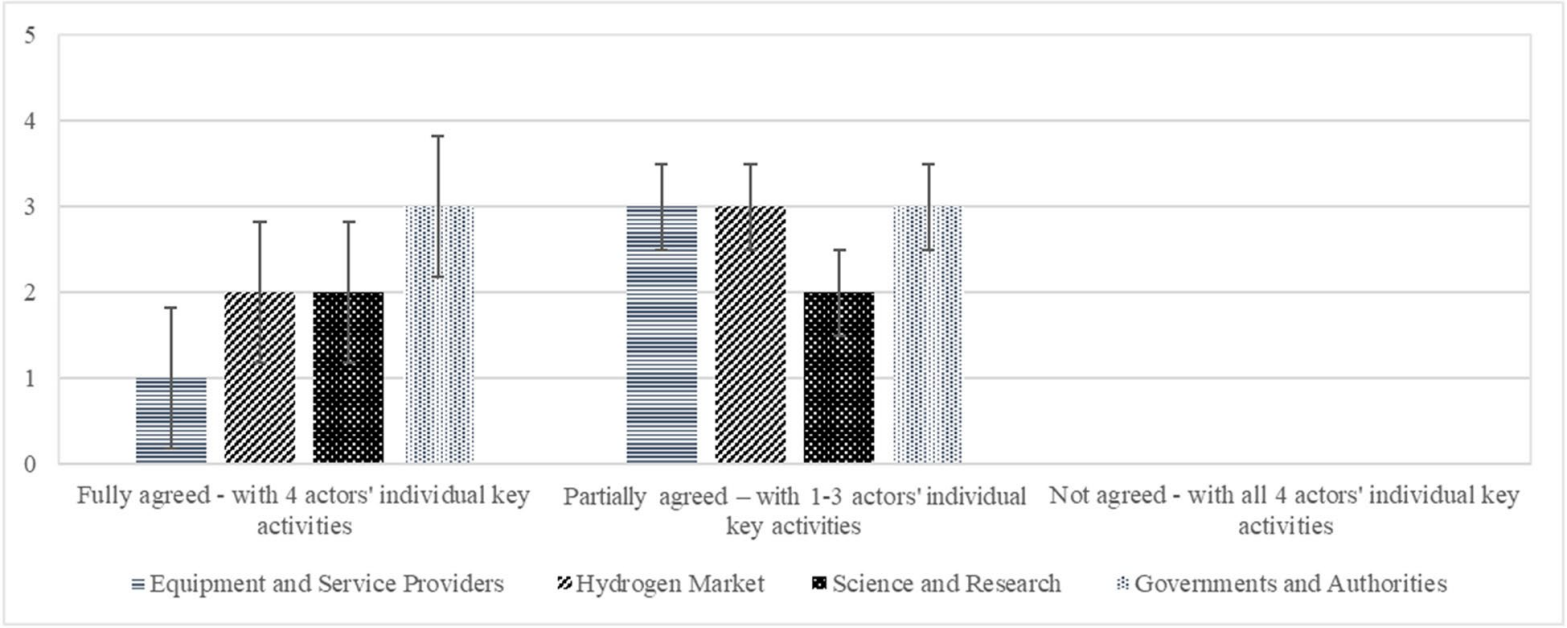
Agreement with predefined key activities. Error bars indicate ± 1 SD. Three abstention

We used a deductive approach to identify key activities, asking about full agreement, partial agreement, and disagreement. The second optional question aims to identify additional actors using an inductive approach. Eight experts agreed and 11 partially agreed (Fig. 7). There were three abstentions. The distribution among the actor segments is not decisive.

The second optional inductive question provides congruent and additional key activities listed in Table 4. These are consistent with the added roles. There are additional mentionings for all four actor segments. Resource flow was assigned to all actor segments except science and research, which is consistent with the results of the ecosystem leadership roles.

**Table 4:** Remarks on actor key activities by experts and focus group discussion participants

Assigned actor segment	Proposed key activities	Remarks on activities by expert interviews (number of mentionings)	Remarks on key activities by focus group discussion
Equipment and service providers	- Assemble components - Supply components - Set-up network - Integrate actors	Congruent: - Set-up networks (1) - Integrate actors (1) Additional: - Generate knowledge (1) - Provide expertise (1) - Transfer technology (1) - Provide complementarities (1) - Orchestrate resource flow (1)	Congruent: - Orchestrate resource flows: coordination of actor segment internal activities - Provide complementarities: certification, standardization - Set-up networks: international network activities - Assemble components: expansion of production Additional: - Give resources: financing projects - Build connection: workflow moderation between the actor segments and central coordination
Science and research	- Generate knowledge - Provide expertise	Congruent: - Generate knowledge: basic research (1), coordination of global and local activities for knowledge generation (1) - Provide expertise: industrial economic and technical research (1), shape economic policy/industrial policy (1), human resources and education (1) Additional: - Transfer technology: develop technology (1)	Congruent: - Provide expertise: education and qualification of future H_2_ and PtX workforce
Governments and authorities	- Create collaboration - Open platforms - Orchestrate resource flows - Orchestrate complementors - Create and capture value - Provide favorable conditions	Congruent: - Orchestrate resource flows: establish contracts for difference for fundings (1) Additional: - Attract and link partners, integrate actors: establish links and integrate other actors (1) - Co-develop offering: co-develop hydrogen and PtX offerings (1) - Define need, provide ideas, purchase, and use: act as state as a consumer of hydrogen (1)	Congruent: - Orchestrate resource flows: market-based incentives (e.g., grants, contract of differences, ensuring green energy on low-cost base) - Attract and link partners: communication to society, showing advantages (e.g., qualification and future jobs) and disadvantages - Provide favorable conditions: attraction of investments, implementing faster approval procedure Additional: - Coordination interaction: regional and global development of joint value creation
Hydrogen market	- Provide complementarities - Purchase and use - Provide ideas - Define need - Build connections - Provide access to markets - Co-develop offerings - Link to other actors	Congruent: - Build connections (1) - Provide access to markets (1) - Provide complementarities: provide infrastructure for transport of hydrogen and PtX derivate (1), stabilize electrical grid (side-effect services) (1), provide evidence/source for CO_2_ involved (1) - Purchase and use: leverage large-scale application for cheap mass production (1) - Co-develop offering: leveraging small-scale solutions for initiation and learning (1) Additional: - Integrate actors (1) - Orchestrate resource flow (1) - Create and capture value: communicate prices to end consumer (1) - Decipher base of value: use opportunity costs for climate instead of appropriate price model (1) -Build platform: system integration into value chain by technology selection (1)	Congruent: - Provide access to markets: traders - Co-develop offering: project developer - Purchase and use: consumer, producing hydrogen and PtX derivates Additional - Give resources: investor

### Focus group discussion

#### Architecture

**Fig. 8:**
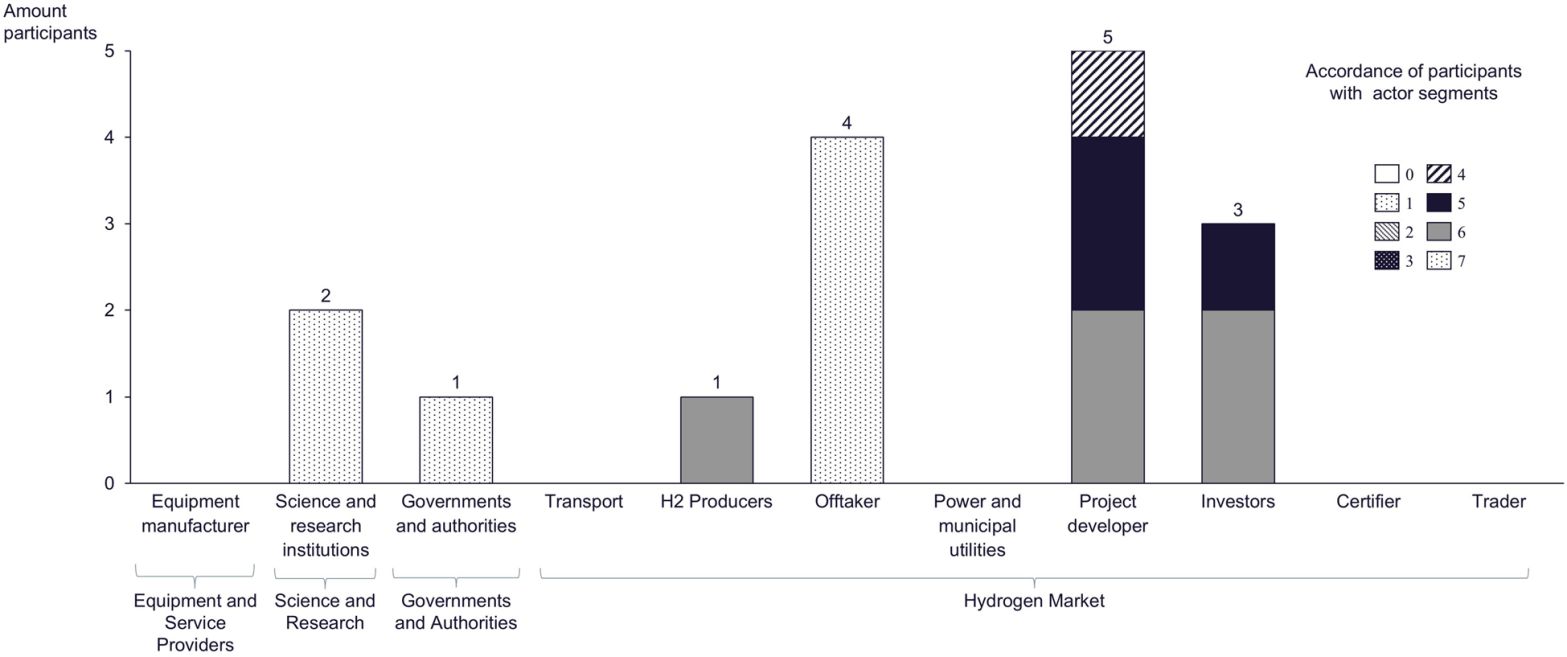
Allocation of participant actor and actor segments

For the first topic of the focus group discussion, groups one and two were asked to assign themselves to the actor categories and the predefined actor segment (Fig. 8). To measure how precise the actors and the segments are defined, a scale from zero to seven was introduced. Most of the participation of groups one and two are from the offtake, the hydrogen market actor segment. The identification is on a bandwidth scale from four to seven. The task was easily solved by the actors. Queries are discussed in the section “Discussion of expert interviews and focus group discussions”.

Although the focus group participants were not directly asked to comment on the actor and actor segments, the majority did. It was pointed out that actors are not allocated properly to the actor segments or missing at all. In general, it was noted that the model is a good starting point, but not specific enough and very general. The high-level view should be more subdivided for a more detailed view. In general, the hydrogen market actor segment was identified as being very diverse in terms of actors, and the differentiation between equipment and service providers was not clear enough. It is noteworthy that there was a discussion about the need for central coordination. This fits with the mentioned key activity of workflow moderation between the actor segments. Remarks from the focus group discussion are captured in Table 5.

**Table 5:** Remarks on the actors and actor segments by the focus group discussion

Actors	Actor categories	Assigned actor segments	Mentionings by the focus group discussion
Original equipment manufacturer	Equipment manufacturer	Equipment and service providers	Fuel cell provider (small scale)
Solution provider, e.g., EPC			
Life cycle service provider			
Suppliers			
Bus manufacturer			
Society (all)	Offtake	Hydrogen market	Society to be considered as actor Agreed view of offtake as part of the hydrogen market
Households			
Mobility (trains, garbage trucks, buses)			
Industry as consumers			
Government authorities (ministries)	Government and authorities	Governments and authorities	Very much local consideration International cooperations and institutions need to be considered, e.g., EU
Gas network operator	Transport	Hydrogen market	Transport and processing could be shifted to equipment and service provider
Storage			
Transporter			
Technical gas companies (processors)			
Producers (with own land)	H_2_ producers	Hydrogen market	Limited amount of producers Market forming depends on the number of actors
Energy company as producer			
Oil and gas companies as producers			
Research institutions Universities	Science and research institutions	Science and research	–
Electricity supplier Grids Electricity producer Water supplier	Power and municipal utilities	Hydrogen market	Could be shifted to equipment and service provider
Project developers Engineering offices	Project developer	Hydrogen market	Agreed view Project developers in hydrogen market
Banks Investors	Investors	Hydrogen market	Banks are very important. Part of the group suggested a shift to equipment and service providers, while another part did not
Platforms Certifier	Certifier	Hydrogen market	Could be shifted to equipment and service provider
Traders Marketer on the exchange	Traders	Hydrogen market	Agreed view traders in hydrogen market
		central expert council (mentioned by focus group discussion)	Associations TÜV

#### Objectives

The answers to the second question, are there any objectives to be added or changed, are shown in Table 2. Decarbonization is seen as an overarching objective and, therefore, mentioned for all actors. That is in line with the identified joint value creation and the underlying common objective decarbonization and comments from the expert interviews. There was no disagreement with the proposed objectives. There are additional mentionings for all actor segments except hydrogen market.

#### Roles

The pre-assigned roles based on the findings from the expert interviews were shared with the focus group participants. In addition to the question of how you would rank the actors for the leadership role, which is not considered in this study, there is the second question of whether there are any actor roles that should be added or changed. The participants did not question the pre-selected roles but made some remarks, as shown in Table 3. For equipment and service providers, a sponsor for banking and a champion for workflow moderation were mentioned. Outside of the four actor segments, a champion role for a central expert council was mentioned.

#### Key activities

The pre-assigned key activities were provided to the focus group participants. The two questions, what could be done differently, and are there key activities that could be added or changed, are answered together. Table 4 shows the result of additional key activities mentioned by the focus group discussion. There are additional comments on the other three actor segments except for science and research.

## Discussion of results of expert interviews and focus group discussion

The explorative mixed-methods study design is the base for researching the actor and actor segments, the objectives, roles, and key activities. That enables the description of the architecture and characteristics of the GHE. The data provided in the “Results” section are discussed in this section.

### Expert interviews

#### Architecture

The expert interview shows that mapping the actor segments along the value chain is the right approach. PtX consumers are mentioned in addition to hydrogen consumers. Since green hydrogen is seen as the basis for all PtX derivatives, the GHE should cover the entire value chain, including PtX products. The hydrogen market group is named relatively frequently with many actors. Thus, the group appears rather large. This could be because the group was not precisely defined at the forefront of the interviews, and the value chain was not shared with the interviewees. If the value chain was considered, the group would be divided along the value chain, which would balance the relationship with the other actor segments. For example, for the equipment and service providers, the original equipment supplier for the renewable energy value chain part will likely be different than for the electrolysis value chain part, and thus its suppliers. For the actor segments, governments and authorities, as well as science and research, the actors along the value chain also change, such as different ministries or different chairs at universities. The triple helix ecosystem described by Russel and Smorodinskaya (2018) with business, knowledge-generating, and public sector seems to be an appropriate model for the foundation of the GHE actor segments.

#### Objectives

The results show a dominant partial agreement and no rejection. The predefined objectives can be regarded as a basic framework. The most frequent disagreements can be seen in the hydrogen market and in the governments and authorities. For the hydrogen market, the objective decarbonization through hydrogen is mentioned for the end users. Furthermore, the aspect of achieving sales and profit is mentioned as well. For governments and authorities, decarbonization is also seen as an important objective besides the realized number of projects. The interviewees revealed that for science and research, the contribution to a better world is in line with decarbonization objective. For equipment and service providers, decarbonization and market strength with a high market share were additionally mentioned. With the objective of decarbonizing, each actor segment is consistent with the overall objective of the GHE. This is in line with the insights gained from the research on joint value creation around the GHE (Schwappach et al. 2024) and with the observation of Cobben et al. (2022). Hydrogen market, equipment and service providers, and governments and authorities currently negatively perceive the relationship between invested effort and objective achievement. Science and research have a positive perception due to the many financial subsidies for research activities related to hydrogen.

#### Roles

The pre-selected roles were fully agreed by 11 experts and partially agreed by 10 experts. There is a tendency towards agreement with the pre-selected roles. The distribution among the actors does not matter. In response to the general question about the roles, it was mentioned that the actor segments equipment and service providers and the hydrogen market are seen as ecosystem leaders in addition to the predefined actor segment governments and authorities. Science and research are not assigned a leading role. The hydrogen market and the equipment and service providers were assigned roles from all four main categories: leadership, direct value creation, value support, and entrepreneurial ecosystem. All mentionings can be considered valid additions due to the experience and knowledge advantage that the experts have in the respective field (Liebold and Trinczek 2009). Both actor segments need to handle the most diverse roles in terms of differences. This transparency and expectation is important because each actor can take on and act the role they want (Thomas et al. 1994). Due to a limited common formal legal structure among actor segments in GHE, decentralized empowerment based on trust is evident (Deiser 2020; Gomes et al. 2018). Governments and authorities actor segment was assigned with roles from three main categories. The actor segment science and research was assigned to roles from one category.

#### Key activities

For the key activities, partial agreement predominates for 11 to full agreement for eight of the experts. Due to the changes in roles previously mentioned, the key activities in the column of additional key activities were adjusted accordingly. In terms of the number of additional key activities mentioned, the equipment and service providers and the hydrogen market again received the most diverse votes from the experts. Overall, it appears that the equipment and service providers and the hydrogen market are at the center of most activities. Government and authorities and science and research seem leaner and limited in the variety of activities. Similar to the roles, key activities can be seen as relevant additions due to the experience and knowledge advantage that the experts have (Liebold and Trinczek 2009). To emphasize the aspect of social acceptance, the government and authorities actor segment is in charge of communicating the benefits of a GHE to society, highlighting the creation of future jobs and qualification measures. Science and research have the key task of training and qualifying future taskforce for H_2_ and PtX. These tasks must be adapted country-specifically and should not be defined in general terms.

### Focus group discussion

#### Architecture

The task of assigning themselves to one of the predefined actor segments was solved by the participants with some clarification questions on the definitions (Fig. 8). Some participants questioned the hydrogen market group of actors, as it appears to be an inclusive segment of actors with a wide variety of actors (Table 5). A proper consideration of the entire value chain would put this statement into perspective. The comment regarding the differentiation between hydrogen market and the equipment and service providers should be reflected by showing the individual actors behind the segments. The suggestion to shift the financing to equipment and service providers could be an understandable step since those actors provide a service to the hydrogen market. On an alternative basis, financing could also be provided via the balance sheet of the equipment and service provider. There are a variety of financing models, e.g., green hydrogen bonds, public–private partnership, venture capital, crowdfunding, impact investment fund, or viability gap funding (Harichandan and Kar 2023). However, the financing models themselves will not solve the underlying financing issue for green hydrogen projects. The financing conditions of green hydrogen projects depend on the country-related risk and the project execution risk (Hydrogen Council and McKinsey & Company 2024). Around 26% of the required financed projects are in the “global south” (Hydrogen Council and McKinsey & Company 2023). Most countries face higher country risks, which turns into high financing costs of additional 4−12% (Hydrogen Council and McKinsey & Company 2024; Coface 2024). The project execution risks are a topic for all projects and are influenced by the availability of long-term offtake agreements, technology maturity, performance guarantees, and purchase agreements with low-levelized costs for electricity (World Economic Forum 2023). Risk-taking is a process that is part of scaling innovation and needs to be addressed by all actor segments, with governments and authorities taking the lead (Adner 2006; Schwappach et al. 2024). The example illustrates that the transition between two segments of actors is possible, and that classification depends on the individual case.

The focus group was brought up the idea of a central expert council actor segment. The focus group discussion debated whether such an intermediary function is needed. Examples for such an actor could be, e.g., Hydrogen Council or International Renewable Energy Agency. The Hydrogen Council was founded by companies mainly from the equipment and service providers and the hydrogen market. It has nearly 150 members from across the hydrogen and PtX value chain (Hydrogen Council 2023). Governments established the International Renewable Energy Agency. In 2022, 168 governments are listed as members of IRENA (International Renewable Energy Agency 2023). Both examples show that there is an inherent desire to strengthen coordination through additional established institutions. Leadership is being provided in part by these organizations. The open question from the ecosystem side is whether these institutions are part of the actor segments of equipment and service providers, hydrogen market, and governments and authorities, since they founded them, or whether they already are the central expert council actor segment discussed by the focus group. In general, it seems advisable to conduct further research of the central expert council.

#### Objectives

The objectives provided for each actor segment were accepted by the participants of the focus group discussion. Additional objectives were added by the participants (Table 2). The participants named the objective of decarbonization for each actor segment. That is consistent with the observation of Cobben et al. (2022) that the overall objective of an ecosystem is in line with actor individuals. Technological leadership was additionally mentioned for equipment and service provider. That is in line with the additional role, the ecosystem leader, identified. As additional objective for science and research, the development of hydrogen and PtX workforce was mentioned. As part of the education system, the role of transferring knowledge to the future workforce can be seen at research institutions and universities. However, it is not limited to these institutions (Faix and Mergenthaler 2015). Competence development is a lifelong subject and is realized through theory acquisition, reality transfer for experience, and reflection by processing the results (Windisch et al. 2021). The social acceptance of hydrogen in society was mentioned by the participants in the focus group discussion as an objective for the government and authorities. For the energy transition in general, social resistance has a direct impact on the costs and timeline for implementation (Bolwig et al. 2020). It can be considered an important objective for governments and authorities. Regional value creation could become a strong argument for social acceptance. A regional network with several locations has a higher chance of jointly creating innovative solutions due to its proximity (Russell and Smorodinskaya 2018; Scaringella and Radziwon 2018).

#### Roles

The pre-selected roles were shared and accepted by the participants. Further roles were assigned to the actor segments (Table 3). The terms used by the focus group discussion are not the ones introduced. The authors transferred the results into the proposed formulation to ensure comparison. The role of the sponsor and champion was additionally mentioned for the equipment and service providers. The used synonym was banking and workflow moderation. For the additional brought-up actor segment central expert council, the role of the champion was identified by using the term intermediary. Champion seems to be the most appropriate description due to the activities “building connections and alliances between actors” and “interacting between partners and sub-segments” (Dedehayir et al. 2018, p. 25).

#### Key activities

The focus group discussion added key activities to all four actor segments (Table 4). Based on the additional mentioned key activity, workflow moderation between the actor segments, the discussion around a central expert council acting as an intermediary was initiated. The driving force appears to be insecurity about the incompletely coordinated key activities. This needs to be further explored. However, dealing with uncertainty should be one of the main activities of all GHE actors, not just a central coordinator’s activity (Vasconcelos Gomes et al. 2018). It is assumed that the equipment and service providers provide resources in the form of financing projects, which is compatible with the role of the sponsor (bank). In addition, workflow facilitation between the actor segments (build connection) is added to the equipment and service providers. This point is in line with the additional role of the champion. Equipment and service providers need to have a close connection to all other actors in the ecosystem to ensure that the products, solutions, and services they develop meet everyone’s requirements. At the same time, they provide feedback on what is feasible from a technical and economic perspective. Particularly in the innovation archetype phase, this contribution to overall coordination in the ecosystem is crucial for defining joint value creation. Governments and authorities should take on coordinating interaction at regional and global levels for joint value creation as ecosystem leaders. To achieve the required volumes of hydrogen and PtX derivatives, a global focus will be necessary. This orientation must be driven by appropriate economic and industrial policies by governments and authorities. However, for governments and authorities, key activities such as coordinating the interaction of regional and global joint value creation development and creating favorable conditions could collide with behavioral politics, legal or bureaucratic obstacles. As these obstacles are country-specific and the study aims to create a holistic (generic) framework for a green hydrogen ecosystem, this aspect cannot be fully considered in the present paper. Giving resources through investment was added as a key activity for the hydrogen market. It can be seen as a useful addition to the key activities in line with the proposed role sponsor.

### Integrative discussion of expert interviews and focus group discussion

This section focuses on the procedure of the two surveys. The quality of both is discussed to determine whether a combination of the results is, in general, considerable. It contrasts the strengths and weaknesses of the two surveys by looking at the area rigor in research, study background, analysis method, credibility, profundity, detail and abundance of findings, and contribution to knowledge based on the framework for quality criteria used in the comparative analysis provided by Atkins et al. (2012). Interestingly, not all participants in the expert survey and focus group discussion adhered to the pre-selected terms and definitions for the objectives, roles, and key activities. This could be due to limited explanation, understanding, and/or coverage of the predefined terms and definitions. As a result, the authors sorted the mentionings into the most appropriate categories. In Table 6, the key aspects of both methods, the expert interview and the focus group discussion, are compared. Area and description were taken over from Atkins et al. (2012, p. 3).

**Table 6:** Comparison of aspects of expert interviews and focus group discussions

Area	Description	Expert interview	Focus group discussion
“Rigor in research conduct”	“Judgement on how carefully the research is carried out; tends to be a judgment of reporting quality”	The experts interviewed were selected according to their belonging to the actor segments. The method used for data collection and analysis was qualitative content analysis as described by Mayring (2015). A semi-structured interview guide was used	The participants in the focus group discussion were not selected by the authors but instead by the organizers of the training. Multiple descriptions were considered for the methodology
“Study context”	“A detailed description is needed to judge wider applicability of the findings; refers to transferability”	The context of the study is the application of the ecosystem concept for green hydrogen. It will demonstrate the applicability and limitations of the methodology. For practitioners, it will provide a guide to lead green hydrogen to success. Since there are only limited studies on the connection between the ecosystem and green hydrogen, an explorative research design is used. Expert interviews are a good start to collecting first opinions on the topic. These initial opinions need to be tested on a larger scale using methods such as discussion groups	
“Analysis procedure”	“An important component of rigor and reliability”	The expert interviews were transcribed by Amberscript (© Amberscript Global B.V.). To ensure quality, the analysis was performed by two researchers. MAXQDA Plus software (© VERBI) was used for the analysis	Data analysis was performed by the first author. No transcript was made. No software was used
“Credibility”	“Judgement on how well the findings is presented and how meaningful or believable they are”	The participants already work in the GHE. They have gathered individual subjective experiences. 22 experts were interviewed. The results were used to fill predefined codes or to identify new codes	Participants want to work in the GHE, or some are already working. They have limited experience with hydrogen. The participants have several years of professional experience, some as leaders. 24 professionals were part of the focus group discussion
“Depth, detail & richness of findings”	“An indication of the quality of the analysis which underlies credibility claims”	There was enough time to consider individual opinions and clarify open questions. The experts’ experiences provided a basis for a deeper dive into the research topic, as the basics are known. The shared experiences from everyday work are personal subjective experiences. The constructive image of the individual reality is captured but not questioned. Interpretation of the results is more complex because there are more contextual messages. The interviewer’s bias is likely to be lower due to the profound knowledge of the experts	Time was too limited to consider all individual opinions and questions. The different knowledge base of the participants did not provide the opportunity to dive deeply into the research topic. Rather, it remained a high-level discussion. The different experiences within and outside the GHE led to inconsistent views on the current state. The plenary discussion provides more freedom to criticize individually constructed reality. An outside perspective could provide an unbiased view of the changes that need to be made. The moderator had a greater influence on the results due to the moderation, the narrower guidance, and the limited basic knowledge of the participants in some cases. Disproportionate speaking time can lead to a distorted view of a dominant speaker
“Contribution to knowledge”	“Judgement on the relevance and potential utility of the findings in relation to policy, practice, or theory How credible are the findings? Are the claims made supported by sufficient evidence?”	Higher relevance for describing the current state. The data helps to understand the consequences of current principles for the existing GHE. It gives practitioners a guide on what to do for leadership in the GHE	The results are evidence of the simplicity of understanding and demand for change in the GHE. The data provide information on whether the model is plausible and comprehensible and what opportunities there are for improvement. The relevance of the outcome may not be as high as the results of the expert interviews, but it is a source of aspects that contribute to the completion of the GHE

Based on the comparison of both surveys, the following logic was used to discuss which of the objectives, roles, and key activities assigned to the segments of actors were accepted for the GHE. Participation of the expert interview and the focus group did not challenge the pre-selected objectives, roles, and key activities. Therefore, the proposed objectives, roles, and key activities are taken as the basis. The additional objectives, roles, and key activities mentioned by the experts were included in the model because of their experience and knowledge advantage (Liebold and Trinczek 2009). It builds the extended basis for further analysis. The additional objectives, roles, and key activities (not listed as proposed by the authors and not mentioned by the experts) mentioned in the focus group discussion are discussed whether they should be considered for GHE model.

## General discussion

**Fig. 9:**
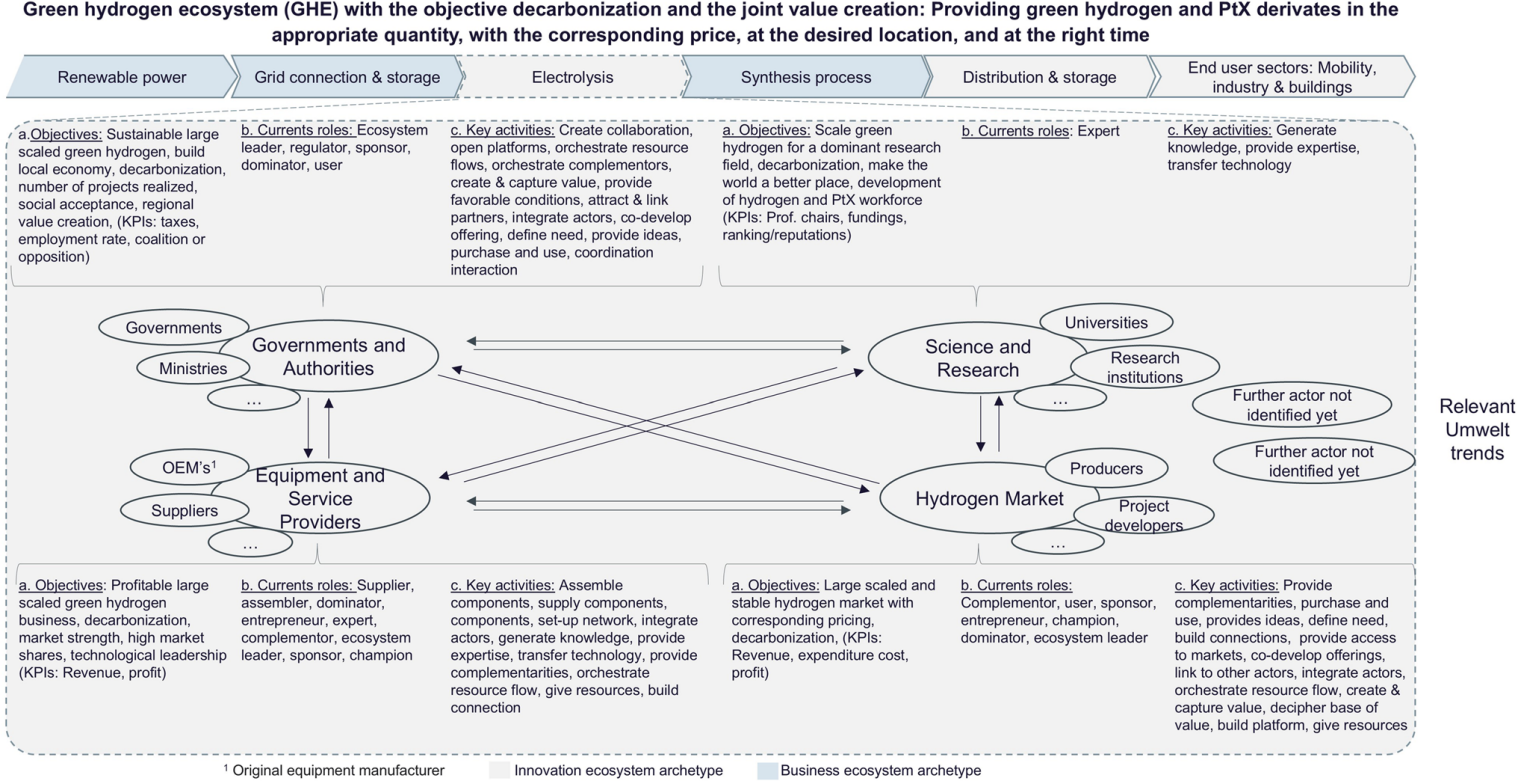
Updated green hydrogen ecosystem (GHE) illustration based on GHE illustration in the section “Illustration” and the insights gained in this paper

In this section, we target to merge the results of expert interviews and focus group discussions into one common picture. As discussed in the previous section, the nature and therewith the quality of both surveys are different. However, we are convinced that the results should lead to a common reality in a discourse on constructive rationality or be classified as an individual subjective reality construct what is not considered as a shared picture. Considering the discussed changes, the sketch of the GHE model is updated in Fig. 9.

For the architecture with the actors within the ecosystem, there are discrepancies in the assignment of actors to the segments of actors mentioned by the focus group discussion. More important than the “right” allocation of actors is the identification of actors to consider all contributors to the joint value creation of the GHE. The transition of actors between the actor segments can be fluid, e.g., equipment manufacturers can also function as owners and operators of equipment, for example, to meet their hydrogen demands and vice versa. Assignment is more of a second priority. A clearly defined characteristic of the segments of actors lends more value to the discussion. The involvement of society is addressed in both surveys. Participation in an ecosystem is defined by an actor’s contribution to joint value creation (Dedehayir et al. 2018). If an actor contributes to the joint value creation, the actor is also part of the ecosystem. Society, in general, does not do this. Rather, it influences the actor segment government and authorities through elections. This is an influence and irritation which, according to Luhmann (2006), is defined as an irritation from the outside of a system. Some individuals from society take hydrogen for their own consumption, for example. These individuals are considered as consumers in the actor segment hydrogen market. The demand for a central expert council raised by the focus group is interesting but was not mentioned by the experts. The question arises as to why the focus group participants make such a demand. This could be based on various aspects, such as fear of the system’s complexity and cultural background. Looking at the principles of ecosystems, it is more of a self-regulating system. The introduction of a central coordinating function leads to the question of whether it is still an ecosystem if a central function intervenes in a regulating way. But on the other hand, such a central actor segment could be the connection between the segments of actors. Provided, this group must be accepted by all actor segments. This requires neutrality. In general, a central expert council can be considered as a suggestion for possible improvement. However, it is recommended that further research be conducted prior to implementation in the GHE model.

For the objectives, a common outcome is that decarbonization is seen as overarching. It applies to all segments of actors. One explanation for this could be that with the Paris Agreement in 2015, most of governments worldwide have committed to decarbonization by limiting the temperature increase to 1.5 (Schumer et al. 2023; European Commission 4/22/2022). This commitment has found its way into various policies and regulations that impact all sectors. It can be assumed that all actor segments are pursuing decarbonization objectives. Additional objectives were mentioned in the expert interviews and the focus group discussion. Other additional mentionings made by the experts in the interview are considered as outlined above. The additional remarks on technology leadership, development of hydrogen and PtX workforce, social acceptance, and regional value creation were discussed in the study and deemed to be useful additions to the GHE. Even if decarbonization has been agreed as a common objective, the pathways to achieve this objective may differ. As mentioned in the example of Australia in the section on the hydrogen background, higher hydrogen consumption will not necessarily contribute to decarbonization (Qadeer et al. 2023). The entire hydrogen circular economy with material extraction, equipment manufacturing, hydrogen production and usage (operation), and recycling must also be considered (Zhang and Zhou 2024). The production and use of hydrogen offers a variety of alternatives as to how the system can be designed and optimized under technical and economic conditions with different CO_2_ emissions (Zhou et al. 2024; Zhou and Zhou 2023). This variety will dive from region to region. For roles and key activities, the proposed and the additions mentioned by the experts are considered for the GHE. For the equipment and service providers, the focus group discussion mentioned the role sponsor using banking and financing projects as an example. This role can be considered as some equipment manufacturers take on the financing of projects. In addition, the role of a champion has been identified for this group of actors, which is in line with the key activities of workflow moderation between the actor segments and central coordination. In combination with the leadership role, the equipment and service providers can be seen as a champion connecting different actors and coordinating some activities centrally for the whole ecosystem by contributing techno-economic expertise. The coordination interaction as key activity is added to governments and authorities. This addition is in line with the pre-selected role of ecosystem leader and is therefore considered for the GHE. For the hydrogen market, the key activity, giving resources as an investor, is consistent with the proposed role sponsor and, therefore, considered in the GHE. The focus group discussion brought up the additional mentioned central expert council actor segment. This segment of actors was discussed above and is not considered. Leadership responsibility is with the actor segments with the lowest return of investment: governments and authorities, equipment and service providers, and hydrogen market. In the medium to long term, this could cause tensions in the ecosystem, as there is an imbalance between the investments in joint value creation and the returns from archiving the objectives of the individual actor segments. In the end, the actors concentrate their resources on other business areas and withdraw from the GHE. This could be remedied by a more transparent allocation and assignment of key activities in relation to objective achievement.

## Conclusion and outlook

This paper investigates into the actors and actor segments, objectives, roles, and key activities as it is needed for describing the architecture and characteristics of the GHE. Two expressions are considered for the illustration. The conducted expert interviews add to the picture of the GHE and provide the basis for the second survey. The focus group discussion is to test the insights gained by the expert interviews. Certain criteria limit the quality of the study. To minimize their impact, it is advisable to conduct interviews and focus group discussions with experts with different professional cultural backgrounds from other markets outside Germany and, in the long term, to set up a panel to monitor changes regularly through qualitative surveys. To gain insights into the practicability of the proposed framework for GHE, the authors encourage readers and users to share their experiences in an inductive format with the first author. The email address can be found on the cover page. Based on the returning feedback, it is recommended to derive a theoretical application framework for the operational transfer of the GHE.

Considering the entire value chain, four actor segments are suitable to describe the main actors. The objectives, roles, and key activities of the actor segments seem appropriate for characterization. Pre-selection is helpful in providing a solid foundation for the interviews and focus group discussion, although subsequent adjustments are necessary. The return of investment, considering the efforts an actor needs to contribute to the joint value creation and the achievement of the actor individual objectives, is currently highest for the actor segment science and research. There are three ecosystem leaders: governments and authorities, equipment and service providers, and hydrogen market. The actor segments with leadership responsibilities have the lowest reward and the most diverse roles and key activities assigned. In the medium to long run, that could cause tension and imbalance in the ecosystem. The focus group discussion revealed the demand for better coordination of key activities between the actor segments. There seems to be a need for a central expert council actor segment. As described, organizations are already attempting to fill this gap.

After defining the architecture and characteristics of the object GHE, as a next step, it is recommended to further study orchestration—ecosystem leadership—and the operations within the ecosystem and interaction with the *Umwelt*. In addition, the influence of *Umwelt* trends to be researched will lead to long-term cycles of iterative adjustments of joint value creation to the *Umwelt* trends (Walrave et al. 2018). The changes in leadership, roles, and responsibilities in the long term are the subject of further research. This will help to understand how leadership in the business ecosystem needs to be adapted to sustain the GHE as it matures. Additionally, the ideas of implementing a central expert council actor segment, if not already covered by organizations like Hydrogen Council or International Renewable Energy Agency, need to be further explored. In general, the interaction between the GHE and the local and global value chain with the associated challenges such as security of supply, tariffs, and trade regulations or the impact of effects of geopolitical tensions with behavioral politics and legal or bureaucratic obstacles is the subject of further research.

The study adds to the framework of the GHE. It helps practitioners better find their place in the ecosystem, and, thus, their ideal contribution and understanding of the position of other actors in the ecosystem. For science, it turns the theoretical ecosystem theory into a real praxis example to learn from. Green hydrogen will help limit greenhouse gas emissions to meet the objectives of the 2015 Paris Agreement. To fulfill this contribution, actors in the GHE must lead the scaling up through innovation.

## Data Availability

The datasets generated and/or analyzed during the current study are not publicly available due to privacy concerns and the sensitive nature of the data involved. However, the data can be made available from the corresponding author on reasonable request and subject to institutional approval, provided that any necessary privacy and confidentiality agreements are in place.

## Appendix

**Table 7:** Questionnaire of interviews

Expressions	Categories	Expert interview, guiding questions
Expression 1: four main segments of actor groups with science and research, hydrogen market, equipment and service providers, and governments and authorities are engaged in the green hydrogen ecosystem	Inductive approach for identification of the actors and actor segments Deductive approach for predefined actor segments: 1. Governments and authorities 2. Science and research 3. Hydrogen market 4. Equipment and service providers	1. What are the actors within the ecosystem for green hydrogen? (2. Can the additional mentioned actors be assigned to 1 of the 4 predefined segments?)
Expression 2: each actor segment is characterized by individual objectives, roles, and key activities, and the leader(s) have the governance visible through their objectives, roles and key activities	Deductive approach for individual objectives of the 4 predefined actor segments: 1. Fully agreed, with 4 actors’ individual objectives 2. Partially agreed, with 1–3 actors’ individual objectives 3. Not agreed, with all 4 actors’ individual objectives Inductive approach for identification of additional objectives Deductive approach for ratio between investment (value contribution) and return (objectives) of invest for each actor: 1. Positive 2. Negative	1. Do you agree with the individual objectives of each of the 4 predefined actors? Please select: fully agreed, partially agreed, or not agreed (2. In case of disagreement, what are the individual objectives?) How would you rate the ratio between investment in contributions to the joint value creation towards your return of investment for your actor segment’s objectives? Please select: positive or negative
	Deductive approach for current roles of 4 predefined actor segments: 1. Fully agreed, with 4 actors’ roles 2. Partially agreed, with 1–3 actors’ roles 3. Not agreed, with all 4 actors’ roles Inductive approach for identification of additional roles Deductive approach for individual key activities of 4 predefined actor segments: 1. Fully agreed, with 4 actors’ key activities 2. Partially agreed, with 1–3 actors’ key activities 3. Not agreed, with all 4 actors’ key activities Inductive approach for identification of additional key activities	1. Do you agree with the individual current roles? Please select: fully agreed, partially agreed, or not agreed (2. In case of disagreement, what are the roles of the 4 predefined actor segments?) Do you agree with the individual key activities? Please select: fully agreed, partially agreed, or not agreed (2. In case of disagreement, what are the individual key activities of the 4 predefined actor segments?)

The prepared visualization of the green hydrogen ecosystem to be shared with the expert. Other actors named by the expert will be included

**Table 8:** Table of interviewees

Number	Actor segment	Position
1	Equipment and service providers	Country Lead for Government Affairs
2	Equipment and service providers	Executive Vice President for Hydrogen Business
3	Equipment and service providers	Vice President Sales
4	Equipment and service providers	Vice President Strategy
5	Equipment and service providers	Chief Technology Expert
6	Hydrogen market	Head of Commercial and Central Task
7	Hydrogen market	Managing Director at Municipal Utility
8	Hydrogen market	Technical Manager Hydrogen
9	Hydrogen market	Business Unit Management
10	Hydrogen market	Chief Technology Officer
11	Science and research	Professor of Power Engineering
12	Science and research	Professor of Digitalized Energy Systems, Head of Energy R&D Division
13	Science and research	Director of the Research Institute for Chemical Energy Conversion
14	Science and research	Professor for Chemistry
15	Science and research	Retired Director Chemical Engineering and Biotechnology Prof. Climate Neutral Chemicals Production Technologies and Processes
16	Governments and authorities	Head of Basic Research and Coordinator at Research Institution
17	Governments and authorities	Politician, Former Parliamentary Group Chairman
18	Governments and authorities	Chairman Association and Federal Commission for Energy Policy of the German Economic Council
19	Governments and authorities	Policy Advisor Senate Department for Economics, Energy
20	Governments and authorities	Director-General, Federal Ministry
21	Governments and authorities	Policy Advisor Senate Economy, Labor, Transport and Digitalization
22	Governments and authorities	Former Member of the German Bundestag, Hydrogen Innovation Officer

## Footnotes

1 von Uexkuell (1909) explored the background of the German term *Umwelt*. Based on animal studies, von Uexkuell explained that an individual’s environment has a significant influence on the individual (Gruevska 2019). The cause-and-effect relationship between an organism and its environment is described by the term *Umwelt*.

2 PtX is a technology for processing electricity into hydrogen by electrolysis of water and, when needed, into other forms of energy such as cooling, heating, liquid fuels, or chemicals (Wulf et al. 2020).
